# Long-term efficacy and safety of low-dose rituximab strategy in neuromyelitis optica spectrum disorder: a retrospective cohort study on treatment compliance and clinical outcomes

**DOI:** 10.1186/s13023-025-03770-9

**Published:** 2025-05-23

**Authors:** Yao Wang, Cong Zhao, Zonghao Pan, Rui Zhang, Yi Li, Hongzeng Li, Daidi Zhao, Jun Guo

**Affiliations:** 1 Department of Neurology, Tangdu Hospital, Fourth Military Medical University, Xi’an, China; 2 Department of Neurology, Air Force Medical Center of PLA, Beijing, China; 3 Department of Neurology, The First Affiliated Hospital of Henan University, Kaifeng, China

**Keywords:** Low-dose, Rituximab, Compliance, Efficacy, Neuromyelitis optica spectrum disorder

## Abstract

**Objective:**

To evaluate the efficacy and safety of low-dose rituximab (RTX) strategy in patients with neuromyelitis optica spectrum disorder (NMOSD) over a period exceeding 5 years and to investigate the impact of treatment compliance on clinical outcomes.

**Methods:**

We conducted a retrospective analysis of 81 NMOSD patients who received low-dose RTX at Tangdu Hospital from January 2014 to December 2019. The treatment protocol involved an induction phase of 100 mg weekly for three weeks, followed by maintenance doses of 100 mg every six months. Demographic characteristics, the expanded disability status scale (EDSS) scores, annualized relapse rates (ARR), number and date of attacks, and adverse events were collected. The influence of compliance on treatment efficacy was assessed using multivariable Cox regression and propensity score-weighted analysis.

**Results:**

Over the follow-up period, 32.1% of patients experienced relapses, with the ARR significantly reduced from a median of 1.5 before treatment to 0 after RTX therapy (*p* < 0.001). EDSS scores improved significantly from a median of 3.5 to 1.5 (*p* < 0.001). The relapse rate was significantly lower in the good compliance group compared to the poor compliance group (9.5% vs. 56.4%, *p* < 0.001), and good compliance was associated with a reduced risk of relapse (HR 0.07; 95% CI 0.02–0.25, *p* < 0.001). A total of 19 patients (23.5%) experienced mild adverse events, with no serious adverse events reported.

**Conclusion:**

Our findings support the long-term efficacy and safety of low-dose RTX strategy in treating NMOSD, highlighting the crucial role of treatment compliance in achieving favorable clinical outcomes.

## Introduction

Neuromyelitis optica spectrum disorder (NMOSD) is a rare autoimmune inflammatory disease affecting the central nervous system. It is characterized by recurrent episodes of optic neuritis and myelitis, and can also manifest with syndromes involving the area postrema, brainstem, diencephalon, and cerebrum [1]. Traditionally mistaken for multiple sclerosis due to overlapping symptoms, NMOSD has now been recognized as a distinct disease, particularly following the discovery of antibodies against aquaporin-4 (AQP4-IgG) [2]. Patients afflicted with NMOSD often experience an aggressive relapsing-remitting disease course, where frequent relapses can result in cumulative damage, leading to lasting disabilities and even mortality. Consequently, immunosuppressive therapies hold paramount importance in preventing relapse and mitigating disability progression.

Due to the crucial pathological role of AQP4-IgG, one of the promising therapies is B-cell depletion. To date, the FDA has approved only one B-cell depletion drug, namely the anti-CD19 monoclonal antibody, inebilizumab, for preventing relapses in NMOSD [3]. Nonetheless, its high cost and limited accessibility have constrained its use in developing countries. Rituximab (RTX), an anti-CD20 monoclonal antibody originally developed to treat B-cell lymphoma, has also been demonstrated to be effective in preventing NMOSD relapses and is widely used off-label globally as a first-line treatment [4, 5]. However, the optimal dosing strategy remains under discussion. In most prior studies, the treatment regimen, characterized as “high-dose”, involved either two intravenous infusions of 1 g of RTX at two-week intervals [6, 7] or a weekly dose of 375 mg/m^2^ over four weeks [8, 9] as induction therapy. Maintenance re-infusions varied from 500 to 1000 mg per cycle and were administered every 6 to 9 months tailored to the clinical functional status and B-cell repopulation. It is undeniable that this high-dose regimen is costly, and occasionally, it may lead to fatal side effects such as progressive multifocal leukoencephalopathy (PML) [10].

Our team previously conducted a study on a low-dose regimen, known as the ‘Tangdu strategy’, which involved induction with 100 mg administered weekly for three consecutive weeks, followed by 100 mg every six months as maintenance therapy [11]. After a median follow-up of 35.5 months, the results demonstrated that the low-dose regimen was both effective and safe [11]. However, there remains a paucity of research on the efficacy of low-dose RTX in NMOSD over a longer follow-up. Additionally, the COVID-19 pandemic has disrupted some patients’ adherence to rigorous maintenance therapy schedules, leaving the effects of the low-dose regimen following treatment interruption yet to be fully understood. In this retrospective cohort study, we aim to assess the therapeutic efficacy and safety profile of an extended course of low-dose RTX therapy in patients suffering from NMOSD for a duration exceeding five years. Additionally, we broadened our analysis to compare outcomes between patients demonstrating good compliance and those exhibiting poor compliance, with the objective of elucidating the impact of adherence on disease prognosis.

## Methods

### Study population

We retrospectively assessed patients with NMOSD who initiated treatment with low-dose RTX at Tangdu Hospital, spanning from January 2014 to December 2019. Inclusion criteria for this study were as follows: (1) A conformed diagnosis according to either the 2006 diagnostic criteria for NMO [12] or the 2015 revised diagnostic criteria for NMOSD [13]; (2) RTX treatment duration, spanning from the initial dose to the final dose, should be a minimum of five years. Exclusion criteria included: (1) NMOSD patients with unknown AQP4-IgG status; (2) a history of prior use of other immunosuppressive agents with less than three months of medication discontinuation; (3) the presence of contraindications or a history of severe or acute allergic reactions to RTX; (4) a diagnosis of other chronic active diseases necessitating immunosuppressive or glucocorticoid therapy; (5) pregnancy, a history of miscarriage, or a declared intention to conceive during the study period. This study was approved by the Ethics Committees of Tangdu Hospital, Air Force Medical University (Fourth Military Medical University) (No. K202212-12).

### Treatment protocol

The low-dose RTX strategy encompassed both an induction therapy and a maintenance therapy as we previously reported^11^. Briefly, RTX was administered at a weekly dosage of 100 mg for three consecutive weeks as an induction therapy. Subsequent to this initial treatment, a maintenance infusion was scheduled to consist of a single 100 mg dose of RTX administered every six-month interval. Rescue therapy for relapses consisted of intravenous methylprednisolone at a dosage of 500 ~ 1000 mg for five consecutive days. In certain cases, patients require re-initiation of RTX induction therapy after experiencing relapses. In principle, all treated patients were required to visit regularly (with an interval of 6 months) and undergo disability status assessment using the Expanded Disability Status Scale (EDSS).

### Clinical assessments and data collection

Patient’s demographic and clinical parameters, including gender, age of onset, serological status (test results for AQP4-IgG), disease duration before first infusion, date of each relapse, date of last follow-up, EDSS score, annualized relapse rate (ARR), initial clinical manifestation, and RTX-related adverse events were collected.

The primary endpoint was clinical relapse after RTX treatment. Clinical relapse was defined as the worsening of new or previous neurological symptoms that lasted at least 24 h, with an increase of EDSS score at least 0.5 points [14]. The secondary endpoints were defined as ARR, EDSS score. ARR was defined as the number of relapses divided by the total observation time.

To assess the influence of compliance on treatment efficacy, we categorized the patient population according to their adherence to the prescribed RTX infusion schedule. Specifically, patients who missed RTX infusions for more than six months twice or more, or for more than twelve months once or more, were categorized as having poor compliance. On the contrary, patients who did not meet the above conditions were designated as having good compliance.

### Statistical analysis

Continuous variables were presented by median and interquartile range (IQR) and were assessed via Mann-Whitney U test. Categorical variables were described by counts and percentages, with chi-squared employed to assess intergroup differences. To investigate the relationship between compliance and disease relapse, overlapping propensity score weighted (PS-OW) analysis was employed to address potential intergroup confounding factors. Firstly, a propensity score for patients with good compliance to RTX was computed using a multivariable logistic regression model, which incorporated variables including gender, age of onset, serum status, disease duration before and after RTX treatment, number of attacks before RTX treatment, EDSS score before RTX treatment, initial clinical manifestation. The selection of variables was based on both clinical expertise and a review of relevant literature [14, 15, 16]. Subsequently, the PS-OW method was applied to determine the weight for each patient which represents the likelihood of that patient being assigned to the opposite group. The standardized mean differences (SMD) between the two groups, both before and after the application of PS-OW, were reported to evaluate whether this method effectively balanced the inter-group bias. A multivariable Cox proportional hazards regression was conducted to compare the risk of relapse following RTX treatment between patients demonstrating good compliance versus those with poor compliance in the unweighted analysis. In the OW-weighted analysis, a univariable Cox proportional hazards regression was used to compare the risk of relapse between the two groups. Kaplan-Meier method with log-rank test was used to compare the relapse-free rate between the good compliance and poor compliance groups. Finally, we conducted a multivariable Cox proportional hazards regression to assess the association of age of onset, disease duration before RTX treatment, number of attacks before RTX treatment and EDSS score before RTX treatment with disease relapse in patients with poor compliance. Statistical analyses were performed using the SPSS version 23.0, R version 4.2.2, and Graphpad Prism 7. A *p*-value of less than 0.05 was considered to have statistical significance.

## Results

### Patient characteristics

**Fig. 1:**
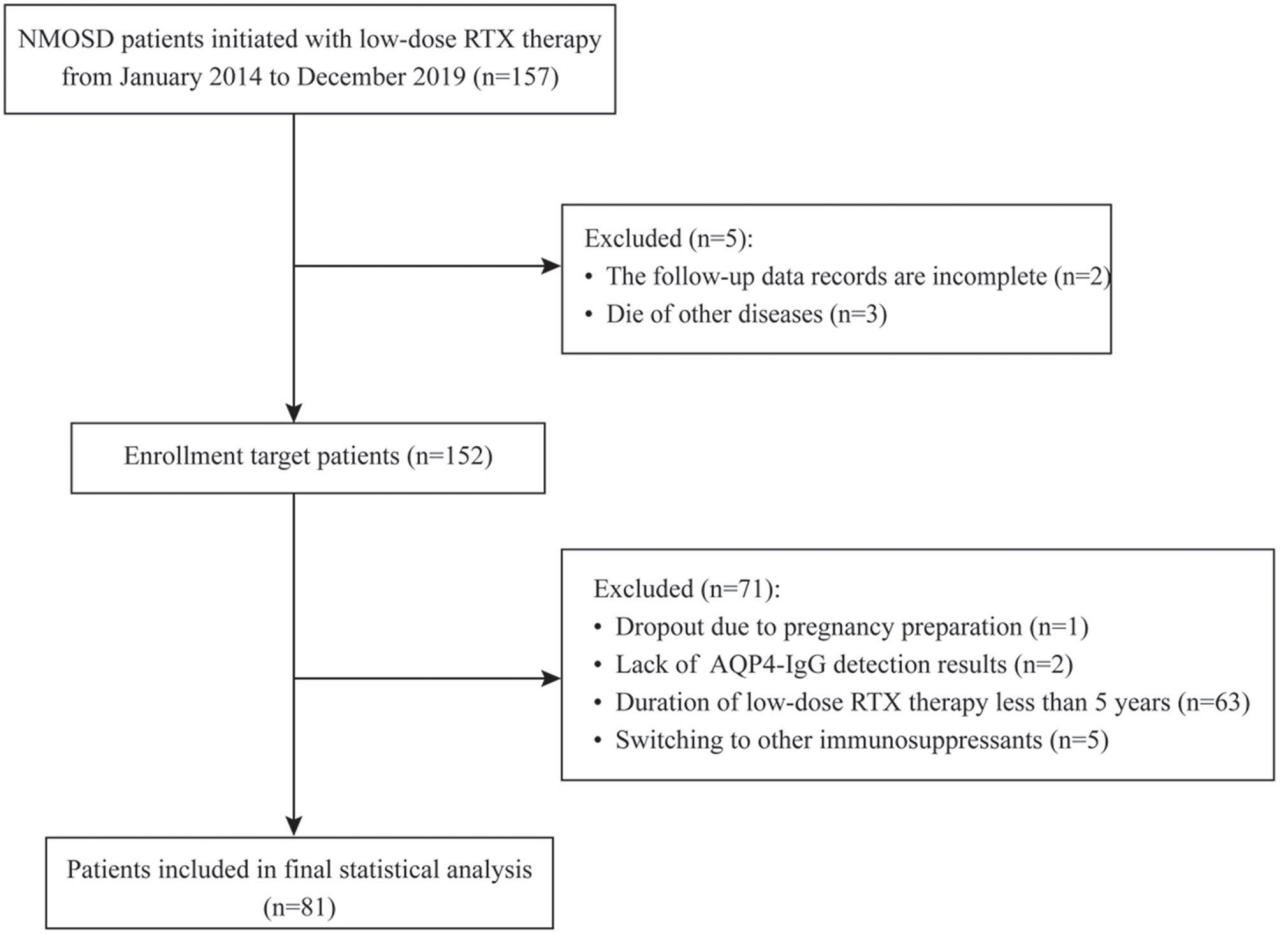
Flowchart of patients enrollment of this study

We identified a total of 157 patients with NMOSD who started low-dose RTX therapy at Tangdu Hospital from January 2014 to December 2019. According to the inclusion and exclusion criteria, 81 patients were included in the final analysis, with the enrollment flowchart shown in Fig. 1.

The demographic and clinical profiles of the 81 patients were summarized in Table 1, comprising 74 females and 7 males. The age of onset for these patients varied from 12 to 66 years, with a median age of 38.0 years. All included patients underwent AQP4-IgG testing. Among them, 70 (86.4%) were seropositive, while 11 (13.6%) were seronegative but fulfilled the 2015 NMOSD diagnostic criteria for antibody-negative cases. The median duration of the disease prior to the initiation of RTX therapy was 6 (IQR, 1–25) months, while the median duration post-RTX therapy was 89 (IQR, 76–100) months. Regarding the clinical presentation at the first attack, myelitis emerged as the predominant phenotype, affecting 58.0% of the patients, succeeded by optic neuritis, which occurred in 28.4% of the cases.

**Table 1:** Baseline characteristics of the 81 NMOSD patients treated with long-term low-dose RTX

Characteristics	Patients receiving long-term RTX (*n* = 81)
Demographics	
Female, n (%)	74 (91.4%)
Age of onset, median (IQR)	38.0 (28.0, 51.0)
Clinical features	
Disease duration of final visit, median (IQR)	100.0 (82.0, 123.0)
Disease duration pre-RTX, median (IQR)	6.0 (1.0, 25.0)
Disease duration post-RTX, median (IQR)	89.0 (76.0, 100.0)
ARR at final visit, median (IQR)	0.3 (0.2, 0.4)
ARR pre-RTX, median (IQR)*	1.5 (0.8, 3.0)
ARR post-RTX, median (IQR)	0.0 (0.0, 0.1)
Serum AQP4-IgG positive, n (%)	70 (86.4%)
EDSS pre-RTX, median (IQR)	3.5 (2.5, 4.0)
EDSS post-RTX, median (IQR)	1.5 (0.0, 3.0)
Initial symptoms	
Optical neuritis, n (%)	23 (28.4%)
Myelitis, n (%)	47 (58.0%)
Brain/brainstem, n (%)	6 (7.4%)
Area postrema, n (%)	14 (17.3%)

Abbreviations: NMOSD, neuromyelitis optica spectrum disorder; RTX, rituximab; IQR, interquartile range; ARR, annualized relapse rate; AQP4, aquaporin-4; EDSS, expanded disability status scale

*To avoid overestimating ARR, 40 patients were excluded because their disease duration before RTX treatment was less than 6 months

### Efficacy of low-dose RTX strategy

**Fig. 2:**
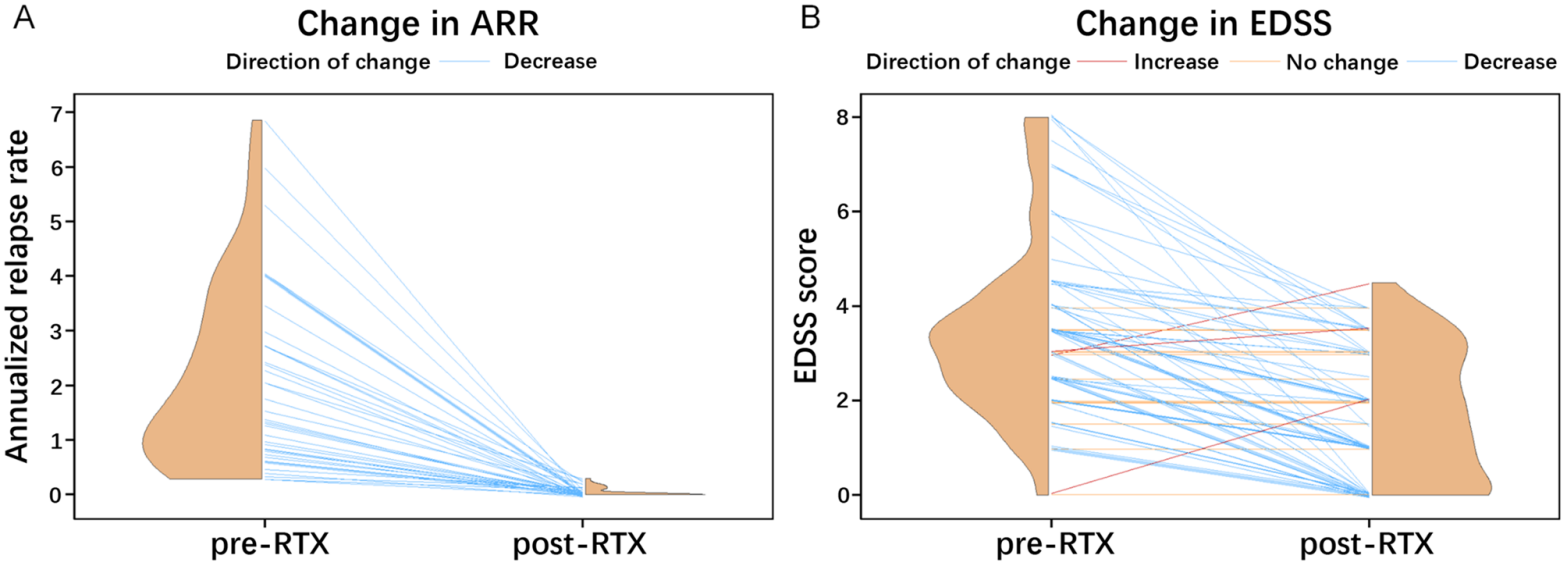
Efficacy of low-dose RTX treatment in patients with NMOSD. (**A**) Changes in annualized relapse rate (ARR) in patients after RTX treatment (*n* = 41). Forty patients were excluded due to a disease duration of less than six months before RTX treatment. Pre-RTX indicates the period from disease onset to the initiation of RTX treatment. Post-RTX indicates the period from initiation of RTX to the last follow-up; (**B**) Changes in expanded disability status scale (EDSS) scores in patients after RTX treatment (*n* = 81). Pre-RTX indicates the time point just before the initiation of RTX treatment. Post-RTX indicates the time point of the last follow-up

A total of 34 relapses occurred in 26 patients (32.1%) during RTX treatment over a follow-up period of no less than 5 years. Meanwhile, 55 patients (67.9%) stayed relapse-free. Figure 2 showed that a reduction in ARR was observed in 41 patients. Before starting RTX therapy, the median ARR stood at 1.5, with an IQR of 0.7 to 2.9. By the final follow-up, the median ARR dropped to 0, and the IQR narrowed to 0 to 0.1, indicating a statistically significant improvement (Z=-5.712, *p* < 0.001). After RTX treatment, the EDSS scores of 63 patients showed a decrease, while the scores of 15 patients remained stable, and the scores of 3 patients increased (Fig. 2). The median EDSS score prior to the initiation of RTX therapy was 3.5 (IQR, 2.5-4.0), which significantly improved to a median score of 1.5 (IQR, 0–3.0) at the final follow-up. This change was statistically significant (Z=-6.724, *p* < 0.001). The decline of ARR and EDSS score suggested a therapeutic benefit of low-dose RTX therapy.

### Comparison between good compliance and poor compliance groups

We grouped the patients based on their compliance with the RTX infusion schedule and compared baseline characteristics and treatment outcomes between the two groups. The two patient groups exhibited disparities in terms of disease duration, the number of attacks prior to RTX therapy, EDSS scores prior to RTX therapy, as well as the proportion of brain/brainstem and posterior area involvement during the first attack. After PS-OW, all baseline characteristics were well balanced between the two groups (SMD < 0.001) as shown in Table 2.

**Fig. 3:**
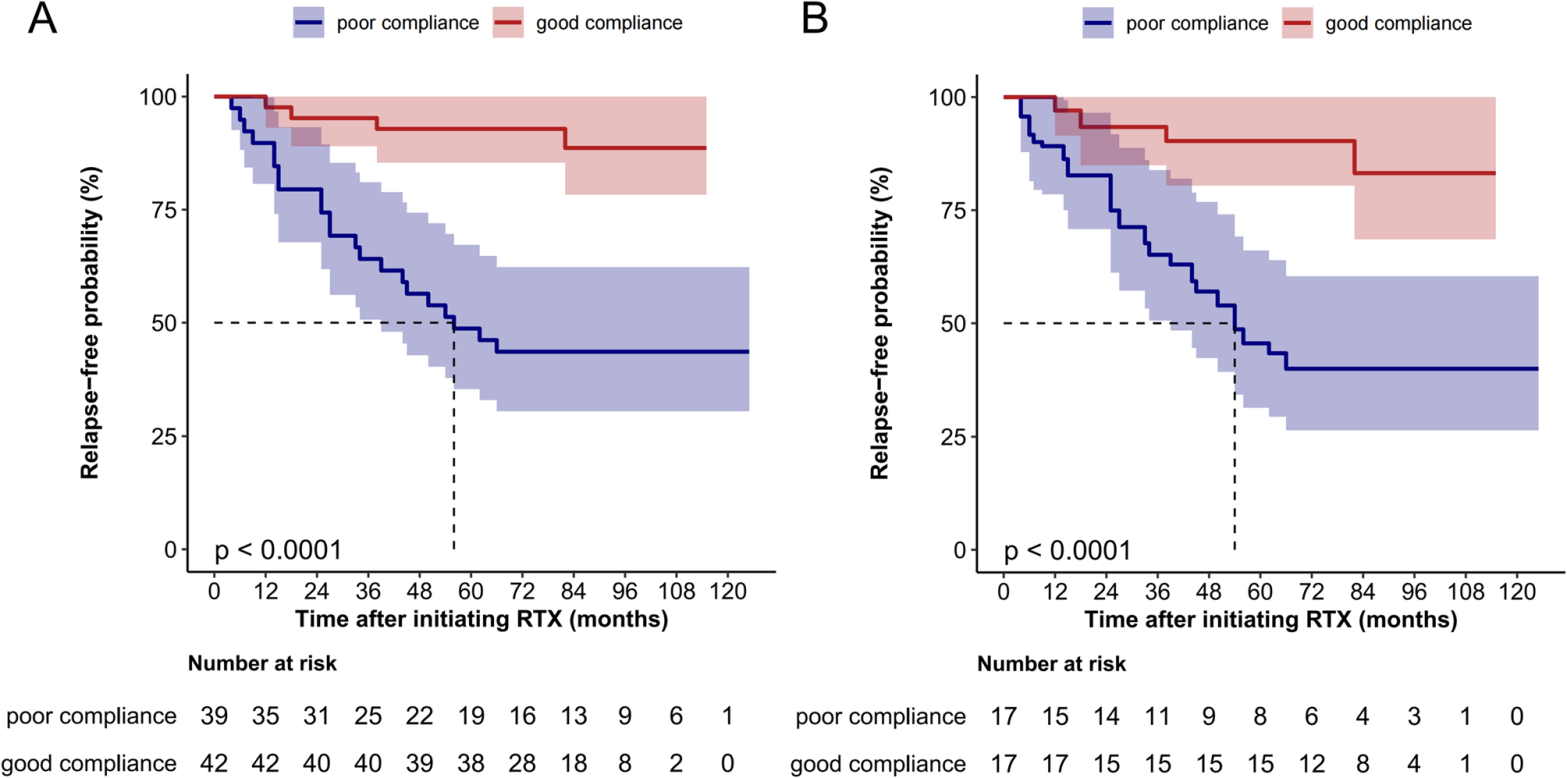
Relapse-free rate analysis in the good compliance and poor compliance groups. (**A**) Kaplan-Meier curves of relapse-free rate in good compliance group (*n* = 42) and poor compliance group (*n* = 39). (**B**) Overlap weighting (OW)-weighted Kaplan-Meier curves of relapse-free rate in good compliance group (*n* = 17) and poor compliance group (*n* = 17)

In the good compliance group, 6 relapses were observed among 4 patients, whereas 22 patients experienced a total of 28 relapses in the poor compliance group. The relapse rate was significantly different between the two groups (9.5% vs. 56.4%, χ^2^ = 20.397, *p* < 0.001). The Kaplan-Meier curve showed that the relapse-free probability was substantially higher in the good compliance group both in the unweighted model and OW-weighted model (Fig. 3). Compared with the poor compliance group, good compliance to the RTX therapy was associated with a lower risk of disease relapse in a multivariable-adjusted Cox model (HR 0.07; 95% CI 0.02–0.25, *p* < 0.001) and a OW-weighted Cox model (HR 0.09; 95% CI 0.02–0.41, *p* = 0.002, see in Table 3). A further multivariable Cox model conducted in patients with poor compliance showed that EDSS scores before RTX therapy was significantly associated with disease relapse in patients with poor compliance (Table 4).

**Table 2:** Baseline characteristics in the unweighted versus overlap weighting (OW)-weighted cohorts

Characteristics	Unweighted			OW-weighted		
	Good compliance (*N* = 42)	Poor compliance (*N* = 39)	SMD	Good compliance (*N* = 16.58)	Poor compliance (*N* = 16.58)	SMD
Female, n (%)	38 (90.5)	36 (92.3)	0.065	15.2 (91.6)	15.2 (91.6)	< 0.001
Age of onset, mean (SD)	38.0 (13.7)	39.6 (13.6)	0.115	38.0 (14.4)	38.0 (13.5)	< 0.001
Serum AQP4-IgG positive, n(%)	37 (88.1)	33 (84.6)	0.102	14.8 (88.9)	14.8 (88.9)	< 0.001
Disease duration pre-RTX, mean (SD)	29.2 (45.9)	18.5 (33.7)	0.265	18.4 (35.6)	18.4 (34.8)	< 0.001
Disease duration post-RTX, mean (SD)	84.3 (15.7)	94.6 (17.7)	0.619	88.9 (16.2)	88.9 (16.3)	< 0.001
Number of attacks pre-RTX, median (IQR)	2.0 (1.0, 4.0)	2.0 (1.0, 3.0)	0.375	1.0 (1.0, 3.0)	2.0 (1.0, 3.0)	< 0.001
EDSS pre-RTX, median (IQR)	3.0 (2.1, 4.0)	3.5 (2.5, 4.5)	0.171	3.1 (2.0, 4.0)	3.5 (2.0, 4.4)	< 0.001
Initial symptoms						
Optical neuritis, n (%)	13 (31.0)	10 (25.6)	0.118	4.7 (28.1)	4.7 (28.1)	< 0.001
Myelitis, n (%)	24 (57.1)	23 (59.0)	0.037	10.1 (60.9)	10.1 (60.9)	< 0.001
Brain/brainstem, n (%)	2 (4.8)	4 (10.3)	0.210	0.8 (4.6)	0.8 (4.6)	< 0.001
Area postrema, n (%)	9 (21.4)	5 (12.8)	0.230	3.0 (17.9)	3.0 (17.9)	< 0.001

Abbreviations: OW, overlapping weighting; SMD, standard mean difference; SD, standard deviation; AQP4, aquaporin-4; RTX, rituximab; EDSS, expanded disability status scale; IQR, interquartile range

**Table 3:** Risk of relapse in patients with good compliance versus patients with poor compliance in the unweighted and overlap weighting (OW)-weighted cohorts

Compliance	Unweighted			OW-weighted		
	Relapse (*n* = 26)	HR (95% CI)*	*P* value	Relapse (*n* = 12.36)	HR (95% CI)**	*P* value
Poor compliance	22 (84.6%)	1 (Ref)		9.96 (80.6%)	1 (Ref)	
Good compliace	4 (15.4%)	0.07 (0.02, 0.25)	< 0.001	2.40 (19.4%)	0.09 (0.02, 0.41)	0.002

Abbreviations: OW, overlapping weighting; HR, hazard ratio; CI, confidence interval; RTX, rituximab

*From Cox proportional hazards model adjusted for sex, age of onset, test results of serum AQP4-IgG, disease duration pre-RTX, disease duration post-RTX, number of attacks pre-RTX, EDSS pre-RTX, and initial symptoms

** From univariate Cox proportional hazards model with PS-OW data

### Adverse events

A total of 19 patients (23.5%) experienced RTX-related adverse events, including infusion reactions and other adverse events (Table 5). The most frequently occurring infusion reaction was skin rash, affecting 7 patients (8.6%), followed by flu-like symptoms (6.2%), skin pruritus (4.9%), perspiration (3.7%), dizziness (1.2%), and laryngeal edema (1.2%). All the infusion reactions were mild and could be quickly relieved through anti-allergic therapy or reducing infusion rates. Other adverse events included alopecia (9.9%), pain (2.5%), urinary infection (1.2%) and leukopenia (1.2%). None of the patients reported serious adverse events such as PML and tumor, and no patients withdrew due to adverse events.

**Table 4:** Risk of relapse in patients with poor compliance

Variables	Unadjusted		Adjusted*	
	HR (95% CI)	*P* value	HR (95% CI)	*P* value
Age of onset	0.99 (0.96 ~ 1.02)	0.578	0.97 (0.93 ~ 1)	0.081
Disease duration pre-RTX	1 (0.99 ~ 1.01)	0.882	1 (0.99 ~ 1.02)	0.75
Number of attacks pre-RTX	0.9 (0.6 ~ 1.33)	0.584	0.69 (0.41 ~ 1.18)	0.175
EDSS pre-RTX	1.2 (0.97 ~ 1.47)	0.088	1.31 (1.03 ~ 1.64)	0.022

Abbreviations: HR, hazard ratio; CI, confidence interval; RTX, rituximab; EDSS, expanded disability status scale

*Adjusted for the four variables

**Table 5:** Low-dose RTX-related adverse events

	Patients with adverse events, *n* (%)
Infusion-related reactions	
Skin rash, n (%)	7 (8.6%)
Flu-like symptoms, n (%)	5 (6.2%)
Skin pruritus, n (%)	4 (4.9%)
Perspiration, n (%)	3 (3.7%)
Dizziness, n (%)	1 (1.2%)
Laryngeal edema, n (%)	1 (1.2%)
Other adverse events	
Alopecia, n (%)	8 (9.9%)
Pain, n (%)	2 (2.5%)
Urinary infection, n (%)	1 (1.2%)
Leukopenia, n (%)	1 (1.2%)

Abbreviations: RTX, rituximab

## Discussion

RTX’s efficacy and safety in NMOSD have led to its recommendation as a first-line treatment, even in off-label use [5]. To date, the RTX dosage regimen still lacks a standardized consensus, leaving the determination of the optimal dosage as a central challenge in the field. Our previous study has demonstrated the efficacy and safety of the ‘Tangdu strategy’, a low-dose RTX regimen featuring weekly 100 mg infusions for three weeks during induction and biannual 100 mg infusions for maintenance in the treatment of NMOSD [11]. However, a significant limitation of our earlier article was the relatively short follow-up duration (with a median follow-up time of 35 months). This was inadequate for evaluating the long-term treatment outcomes extending beyond five years as outlined in a previous article [18]. Furthermore, our previous article was a rigorously designed prospective study, but in the real-world setting patients did not strictly adhere to the prescribed RTX infusion schedule due to various reasons (such as financial constraints, the pandemic, transportation issues, etc.). Therefore, the effectiveness of our regimen in the real world, as well as the impact of patient compliance on treatment outcomes, remained unknown. This prompted us to carry out the current retrospective study. In this study, 81 patients with NMOSD were treated with ‘Tangdu strategy’ for a period exceeding five years. The key findings of this study are as follows: (1) The efficacy of our low-dose RTX therapy was demonstrated to be enduring, with a marked reduction in ARR and an improved EDSS scores, indicating sustained therapeutic benefits over an extended period of more than 5 years. (2) This dosing strategy was characterized by a favorable long-term safety profile, with a reduced incidence of infusion-related reactions, infections, and other serious adverse events. (3) The diminished cumulative risk of relapse among NMOSD patients was positively correlated with the adherence to a scheduled RTX therapeutic regimen, thereby highlighting the importance of strict compliance with the treatment protocol.

The therapeutic efficacy of RTX in the management of NMOSD has been extensively validated since Cree et al.’s report in 2005 [19]. Despite this, most prior research favored a high-dose RTX induction protocol, which entails either 375 mg/m^2^ administered weekly over four successive weeks or 1000 mg administered in two doses separated by two weeks. Maintenance dose of 500 to 1000 mg were typically administered at intervals of 6 to 9 months based on the re-population of B cells [20]. This therapeutic regimen is inspired by the treatment protocol for B-cell lymphoma, which is marked by its high dosages, substantial costs, and the potential for severe adverse effects, including PML. During the last decade, several low-dose RTX strategies has been employed to treat NMOSD [20, 21, 22, 23, 24] and other autoimmune disease [26, 27], demonstrating both good efficacy and a safety profile. Compared with the low-dose RTX studies mentioned above, the dosage used in our study was significantly much lower. Surprisingly, our extremely low-dose RTX regimen resulted in a 100% reduction in the median ARR and a 57.1% reduction in the median EDSS scores over a period of more than five years. Moreover, 67.9% patients remained relapse-free, which is similar to those of studies using standard or low-dose RTX strategies [22, 28]. Our research findings are also consistent with the recommendations of a recent meta-analysis, which suggested that these two regimens, namely 100 mg per week for 3 consecutive weeks and 1000 mg twice with a two-week interval, were more effective in reducing ARR and EDSS [29]. So, we believe that the ‘Tangdu strategy’ employed in this study offers a promising option for the long-term management of NMOSD.

During the follow-up, some patients exhibited poor compliance and did not receive RTX treatments as scheduled. This non-adherence may have been driven by the economic burden and lack of confidence in the efficacy of treatment. Additionally, the COVID-19 pandemic occurred during the follow-up period, further exacerbating the issue by causing disruptions in healthcare access and increasing patient anxiety, which contributed to a further decline in treatment adherence for some individuals. One of the central findings of this study is the strong correlation between treatment adherence and clinical outcomes. Compared to patients with poor adherence, the proportion of relapsers among patients with good adherence was significantly lower. Additionally, both the multivariate Cox proportional hazards regression and the OW-weighted Cox regression analyses collectively indicated that good adherence had a significant protective effect against disease relapse. Our results were in consistent with a previous study which indicated that increased infusion intervals (from 6 months to 14 months) were associated with disease reactivation [15]. Additionally, our research findings indicated that baseline EDSS scores were significantly associated with disease relapse in patients exhibiting poor compliance. These underscored the urgent need to reinforce compliance education, especially targeted at patients with high EDSS scores. To the best of our knowledge, our study is the first to underscore the significance of treatment compliance, thereby emphasizing the necessity of regular follow-up and robust patient education, which might play a pivotal role in preventing disease progression and reducing the risk of long-term disability.

As for the safety profile, our low-dose RTX therapy was notably well tolerated throughout this study. Notably, all of the adverse events were classified as mild to moderate and reversible. No serious adverse events were recorded, including leukopenia, severe infections, malignancies, or cardiovascular complications. Overall, the low-dose RTX regimen evaluated in this study demonstrates a robust safety profile.

Several limitations of this study should not be overlooked. Firstly, the retrospective design of this cohort study inherently limits the ability to establish causality between long-term low-dose RTX therapy and clinical outcomes. A multi-center randomized controlled trial is needed to compare our low-dose RTX strategy with other disease-modifying therapies for NMOSD. Secondly, the ARR before RTX therapy was not applicable in 49.4% (40 out of 81) of patients because they had a disease duration of less than six months prior to their first RTX infusion. Consequently, these patients were excluded from the comparison of the ARR before and after RTX therapy. Future studies with comprehensive data for all participants are necessary to draw more reliable conclusions. Thirdly, the sample size may limit the generalizability of the findings. Although our cohort included a significant number of patients, the diversity of the population may not fully represent all patients with NMOSD. There are still some significant differences in some baseline characteristics between the good compliance group and poor compliance group. Larger multicenter studies are needed to validate our results across different demographics and clinical settings. Finally, the assessment of treatment compliance relied on patient reports and medical records, which may not capture the full picture of adherence behaviors. Factors such as patient motivation, understanding of the disease, and socio-economic influences could significantly impact compliance but were not comprehensively evaluated in this study.

## Conclusion

Our findings support the long-term efficacy and safety of low-dose RTX in the treatment of NMOSD, with significant reductions in ARR and EDSS scores over time. Adherence to the prescribed RTX regimen is critical to achieving these clinical benefits, and low-dose RTX offers a favorable safety profile. Future randomized controlled trial studies are needed to validate these results and further explore the optimal dosing strategies for RTX in NMOSD.

## Data Availability

The datasets that were generated and/or analyzed throughout the course of this study can be obtained from the corresponding author upon receipt of a reasonable request.

## Abbreviations

RTX: Rituximab
NMOSD: Neuromyelitis optica spectrum disorder
EDSS: Expanded disability status scale
ARR: Annualized relapse rate
AQP4: Aquaporin-4
PML: Progressive multifocal leukoencephalopathy
IQR: Interquartile range
PS: Propensity score
OW: Overlapping weighted
SMD: Standardized mean differences
